# Apelin attenuates the osteoblastic differentiation of aortic valve interstitial cells via the ERK and PI3-K/Akt pathways

**DOI:** 10.1007/s00726-015-2020-3

**Published:** 2015-07-05

**Authors:** Zhao-shun Yuan, Yang-zhao Zhou, Xiao-bo Liao, Jia-wen Luo, Kang-jun Shen, Ye-rong Hu, Lu Gu, Jian-ming Li, Chang-ming Tan, He-ming Chen, Xin-min Zhou

**Affiliations:** Department of Cardiovascular Surgery, The Second Xiangya Hospital, Central South University, Changsha, 410011 Hunan People’s Republic of China

**Keywords:** Aortic valve calcification, APJ, Alkaline phosphatase, Runt-related transcription factor 2, Mineralization

## Abstract

Aortic valve calcification (AVC), which used to be recognized as a passive and irreversible process, is now widely accepted as an active and regulated process characterized by osteoblastic differentiation of aortic valve interstitial cells (AVICs). Apelin, the endogenous ligand for G-protein-coupled receptor APJ, was found to have protective cardiovascular effects in several studies. However, the effects and mechanisms of apelin on osteoblastic differentiation of AVICs have not been elucidated. Using a pro-calcific medium, we devised a method to produce calcific human AVICs. These cells were used to study the relationship between apelin and the osteoblastic calcification of AVICs and the involved signaling pathways. Alkaline phosphatase (ALP) activity/expression and runt-related transcription factor 2 (Runx2) expression were examined as hallmark proteins in this research. The involved signaling pathways were studied using the extracellular signal-regulated kinase (ERK) inhibitor, PD98059, and the phosphatidylinositol 3-kinase (PI3-K) inhibitor, LY294002. The results indicate that apelin attenuates the expression and activity of ALP, the expression of Runx2, and the formation of mineralized nodules. This protective effect was dependent on the dose of apelin, reaching the maximum at 100 pM, and was connected to activity of ERK and Akt (a downstream effector of PI3-K). The activation of ERK and PI3-K initiated the effects of apelin on ALP activity/expression and Runx2, but PD98059 and LY294002 abolished the effect. These results demonstrate that apelin attenuates the osteoblastic differentiation of AVICs via the ERK and PI3-K/Akt pathway.

## Introduction

Aortic valve stenosis (AS) is the most frequent type of valvular heart diseases (VHD) in Europe and North America, and calcific AS is the primary presentation in adults of advanced age (2–7 % of the population >65 years of age) (Vahanian et al. 2012; Iung et al. 2003; Nkomo et al. 2006). Calcific aortic valve diseases (CAVD) are characterized by the deposition of calcified plaques, thickening and rigidity of the leaflets, and are increasingly affecting the aged population. Many factors contribute to the risk of the disease, including age, sex, tobacco use, hypercholesterolemia, and hypertension, all having worldwide distribution (Rajamannan et al. 2011).

Aortic valve calcification (AVC) used to be described as degenerative and irreversible (Sell and Scully 1965). Currently, surgery is the only effective option for patients with calcific AS, since the mechanisms of valve calcification are poorly understood and the non-surgical options are not defined. However, many recent studies suggest AVC is not a passive, degenerative, irreversible aortic valve disease, but the result of an active, regulated and reversible progression in the pathogenesis of calcification, including the osteoblastic differentiation of aortic valve interstitial cells (AVICs) and resulting osteogenesis (Mohler et al. 2001; Rajamannan et al. 2003). We have provided evidence that AVC resembles osteogenesis, and that AVICs play a crucial role in this process (Feng et al. 2012). The osteoblastic differentiation of AVICs is the core mechanism ofAVC. As we described previously, AVICs function both as fibroblasts, secreting collagen and shaping the extracellular matrix, and as smooth muscle cells contracting according to the tension of the aortic valve. In pro-calcific medium (PCM) containing dexamethasone, AVICs differentiate into osteoblasts capable of calcium deposition and express markers of osteoblast formation runt-related transcription factor 2 (Runx2) and alkaline phosphatase (ALP).

Apelin, a 77 amino acid pre-pro-peptide, was first discovered by activating the G-protein-coupled receptor APJ (Tatemoto et al. 1998). Apelin can be cleaved into active fragments of various sizes. The major active forms have 13 to 36 residues, such as apelin-13. APJ, the apelin receptor, was first described as a receptor resembling AT1, angiotensin-I receptor (O’Dowd et al. 1993), and is a member of the rhodopsin family (Fredriksson et al. 2003) of heptahelical G-protein-coupling receptors, also known as family A of these receptors (Horn et al. 1998). APJ is widely distributed in the cardiovascular system, myocardial cells, endothelial cells, and vascular smooth muscle cells (VSMCs) (Hosoya et al. 2000; Kawamata et al. 2001; Kleinz and Davenport 2004; Kleinz et al. 2005).

In recent years, the apelin/APJ pathway was demonstrated as a potent regulator of cardiovascular function, mediating adaptation to physiological stress and disease (Lee et al. 2006; Quertermous 2007; Chandrasekaran et al. 2008; Peltonen et al. 2009; Shan et al. 2011; Cui et al. 2010), including contractility in cardiomyocytes, proliferation and myosin light chain phosphorylation in VSMCs, and inhibition of calcification and apoptosis of VSMCs. This encouraged us to investigate how apelin/APJ act in AVC. Apelin was found to be expressed in aortic valvular endothelial cells, with lower expression in calcific valves, where the apelin-APJ signaling pathway was found to be significantly up-regulated (Peltonen et al. 2009). However, the exact mechanism of apelin in AVC, and specifically which occurs in AVICs, has not yet been elucidated.

The present study is based on hypothesis that the apelin/APJ signaling system is involved in the osteoblastic differentiation of human AVICs. To explore the effect of apelin on the osteoblastic differentiation of human AVICs and the potential mechanisms, we induced calcification of cultured using pro-calcific medium containing dexamethasone (DXM), and examined the potential cell signaling pathways involved in osteoblastic calcification as related to treatment with apelin. The results show that apelin can directly regulate the osteoblastic differentiation of human AVICs via the extracellular signal-regulated kinase (ERK) and phosphatidylinositol 3-kinase (PI3-K)/Akt signaling pathways. This new evidence provides a novel preventive and therapeutic strategy for AVC.

## Materials and methods

### Isolation and culture of cells

The aortic valves were collected by Bentall surgery at the Second Xiangya Hospital of Central South University, from patients having the Stanford type A aortic dissection. The patients (mean age 30 ± 7.5 years) had no previous heart valvular diseases. The tiny lesions of aortic valvular leaflets were removed during the pathological examination. This study was approved by the ethics committee of the Second Xiangya Hospital, and all patients provided informed consent.

The isolation and culture of AVICs was performed as previously described (Feng et al. 2012; Meng et al. 2008), with some modifications. The distal 2/3 of the aortic valve leaflets were collected in the operating room and rinsed in phosphate buffer saline (PBS) with penicillin G/streptomycin (200 U/ml). The leaflets were finely minced (1 × 1 mm) and then digested with 2.5 mg/ml type II collagenase and 0.125 % trypsin with 0.02 % EDTA for 90 min at 37 °C, with vortexing for 10 s every 15 min. The softened leaflet fragments with free cells were centrifuged at 1000 g for 5 min. Then, the supernatant was discarded and the precipitate was blended in full medium [Dulbecco’s Modified Eagle’s Medium (DMEM, Hyclone with 4.5 g/L glucose) with 20 % fetal bovine serum (Hyclone) and penicillin G/streptomycin)]. Finally, cells were plated onto six-well plates and cultured in a cell culture incubator supplied with 5 % CO_2_. Upon reaching 80–90 % confluence, the cells were sub-cultured in 75 cm^2^ flasks, and passages 3–6 were used for experiments. To ensure that the cultured cells were not contaminated with other cells throughout the process, we had detected SM α-actin, vimentin, SM-myosin, desmin and CD31 by immunohistochemical method as described in our previous study (Feng et al. 2012).

### Expression of APJ in cultured human AVICs

To check for expression of APJ in human AVICs, western blotting and immunohistochemical method were performed. The cells cultured in a 48-well plate were washed with PBS, and fixed in 4 % paraformaldehyde for 5 min. Cells were blocked by non-immune goat serum and incubated with antihuman APJ (Proteintech) overnight at 4 °C. The cells were then washed with PBS and incubated with goat anti-rabbit antibody conjugated with biotin (Beyotime) for 20 min at room temperature. Following that, the cells were washed by PBS and incubated with streptavidin-conjugate horseradish peroxidase. Cells were then stained by the AEC method. Stained cells were visualized using a Nikon TE300 phase contrast microscope.

### Alkaline phosphatase activity

The AVICs at about 90 % confluence in 48-well plates were divided into six groups. These groups were treated with full medium, PCM [containing 10 mM beta-glycerophosphate disodium (β-GP), 10 nM dexamethasone (DXM) and 50 μg/ml ascorbic acid (Vit C)], and PCM with four different concentrations of apelin every other day. The first day of culture in the supplement was defined as day 0. On day 21 of the experiment, ALP activity was detected by the 4-chloro-3-indolyl phosphate/nitroblue tetrazolium chloride (BCIP/NBT) method.

### Osteoblastic differentiation of cells

The AVICs at about 90 % confluence in 24-well plates were divided into three groups, which were, respectively, treated with full medium, PCM and PCM with 100 pM apelin every other day. The first day of culture in PCM was defined as day 0. The calcification of cells was detected on day 21 by von Kossa staining. Then, quantification of calcium content in calcified plaques was performed as previously described (Feng et al. 2012). The three groups of cells were decalcified with 0.6 M HCL for 24 h. The calcium content was determined by measuring the concentrations of calcium in the supernatant by atomic absorption spectroscopy. The remaining cells were washed with PBS for three times and lysed in 1 × cell lysis buffer with complete EDTA-free protease inhibitor. The total protein content was measured by the BCA method, and the calcium content was normalized to total protein content.

### Western blotting

AVICs in 6-well plates were lysed in 1 × cell lysis buffer (Beyotime) with complete EDTA-free protease inhibitor (Roche). The protein content was detected using the bicinchoninic acid (BCA) method. Denatured protein samples with 1 × loading buffer were separated by 12 % polyacrylamide gel electrophoresis followed by transfer to a pure nitrocellulose blotting membrane (Pall Corporation). Proteins of interest (such as APJ, ALP, Runx2) were then blotted using antihuman antibodies (Proteintech), horseradish peroxidase conjugate second antibody (Proteintech) and Western blot chemiluminescence detection (Millipore). The expression of target protein was normalized to β-actin.

### Detection of mitogen-activated protein kinase (MAPK) and PI3-K/Akt

Cultured cells in 6-well plates were first treated with 100 pM apelin for 0, 5, 15, 30, 60 or 120 min. The treated cells were also lysed in 1 × cell lysis buffer (Beyotime) with complete EDTA-free protease inhibitor (Roche). The BCA method was used to determine the protein content. Western blotting was performed as above. Proteins were then transferred to a nitrocellulose membrane. The membrane was incubated with ERK, phosphate-ERK, p38, phosphate-p38, c-Jun N-terminal kinases (JNK), phosphate-JNK, Akt, or phosphate-Akt antibodies (Santa Cruz) at 1:1000 dilution overnight. The membrane was then incubated with goat anti-mouse IgG antibody or rabbit IgG/horseradish peroxidase conjugate at 1:5000 in PBS for 1 h. Blots were processed using an ECL kit and exposed to X-ray film.

### Data presentation and statistics

All data are presented as mean ± standard error. Comparisons among values of more than two groups were performed by one-way ANOVA. A *P* value below 0.05 was considered significant.

## Results

### Human AVICs express APJ

**Fig. 1:**
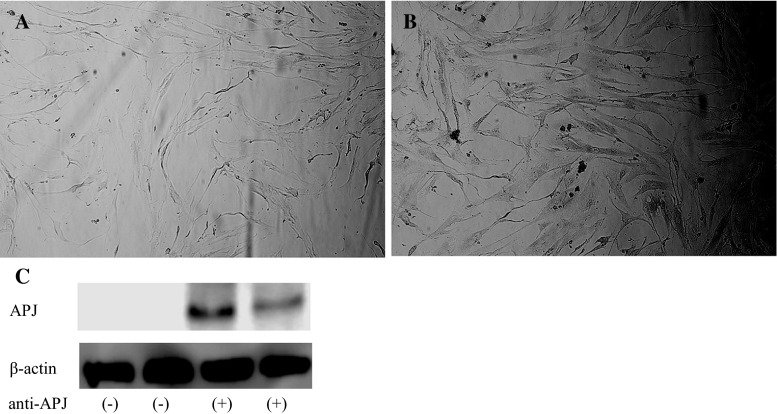
The expression of APJ in AVICs. Staining of cultured AVICs. **a** Negative control incubated with PBS, **b** APJ positive incubated with anti-APJ. **c** Western blotting shows APJ positive incubated with anti-APJ

Using immunohistochemistry, we found that human AVICs expressed APJ. To further confirm the result, western blotting was also performed. As seen in Fig. 1b, and the last two lanes in Fig. 1c, APJ expression was positive. The PBS negative control is shown in Fig. 1a and the first two lanes in Fig. 1c.

### Apelin attenuates osteoblastic differentiation and mineralization of human AVICs

Calcific aortic valve expresses some bone-related molecules, such as osteopontin (OPN), osteoprotegerin (OPG), ALP, and Runx2, and the valve calcification process resembles the bone mineralization process (Mohler et al. 2001). To identify the effect of apelin on the osteoblastic differentiation and mineralization of human AVICs, we supplemented the cells in PCM with apelin at different concentrations, and examined two important osteoblastic markers, ALP and Runx2, as well as mineralization.

**Fig. 2:**
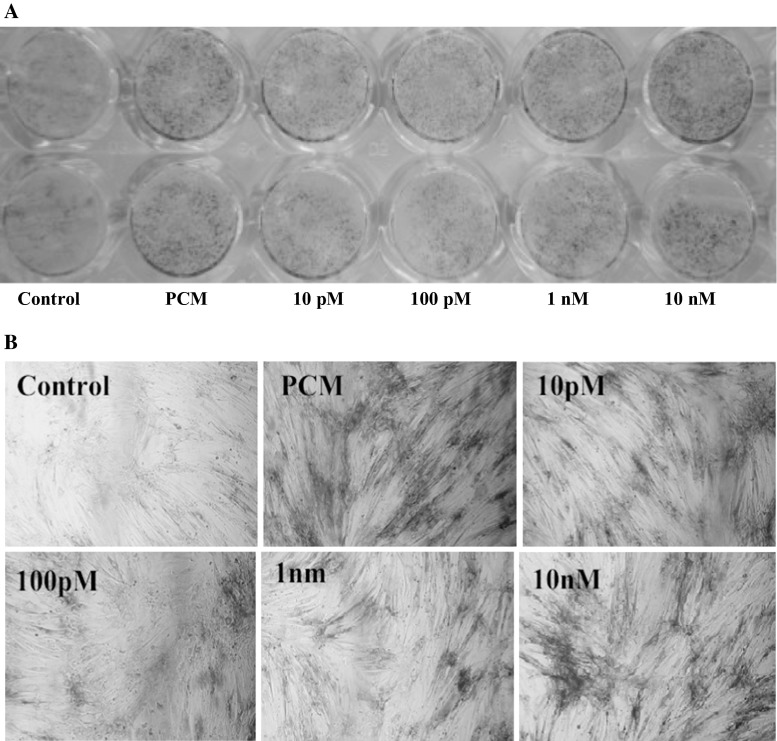
The effect of apelin on ALP activity. Human AVICs were induced with PCM containing apelin at different concentrations for 14 days. **a** A representative view of the BCIP/NBT staining method in 48-well plates. **b** A representative microscopic view of the BCIP/NBT method staining at a magnification of ×400 in 48-well plates

**Fig. 3:**
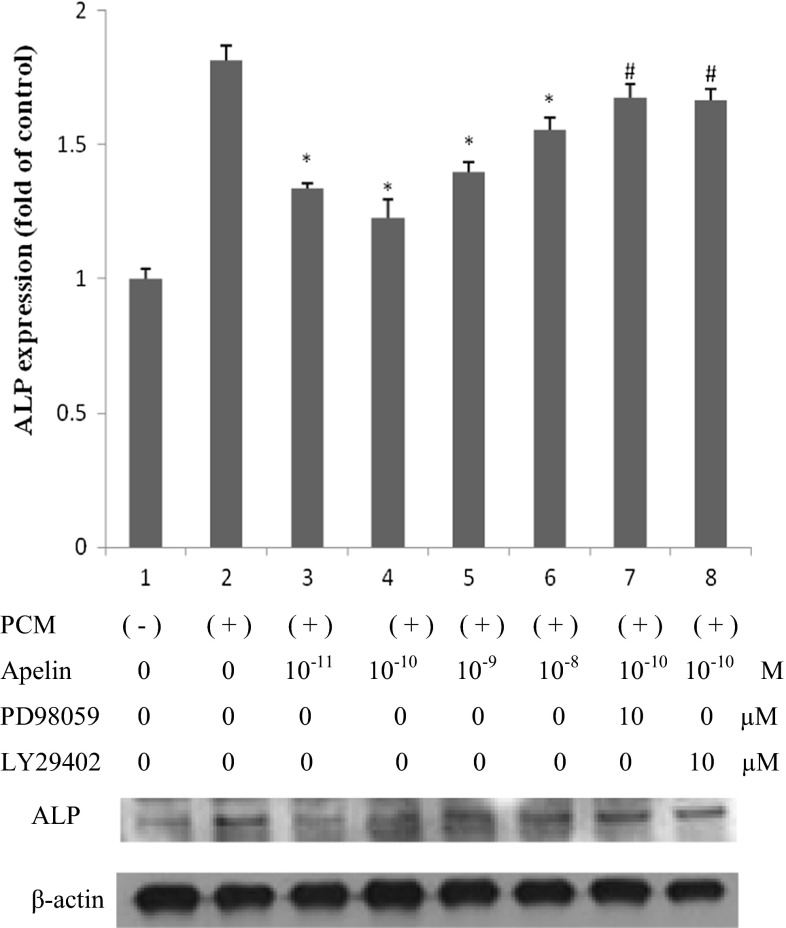
The effect of apelin on ALP expression. Human AVICs were induced with PCM containing apelin at different concentrations (0, 10 pM, 100 pM, 1 nM and 10 nM) as well as PD98059 (ERK inhibitor) and LY29402 (Akt inhibitor) for 14 days

**Fig. 4:**
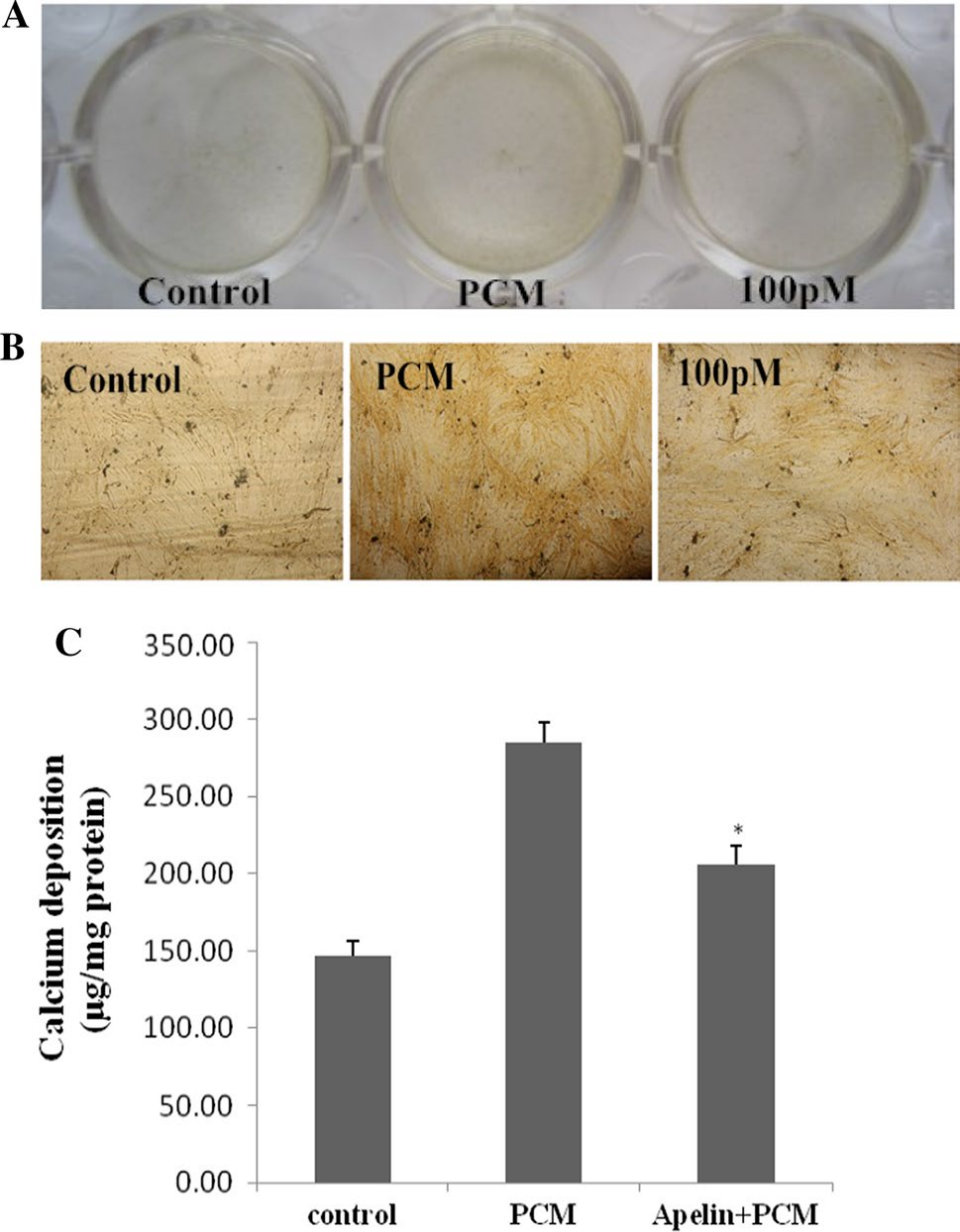
The effect of apelin on AVIC mineralization. Human AVICs were incubated in vehicle, PCM and PCM containing 100 pM apelin for 21 days. **a** A representative entire plate view of the von Kossa staining method in 24-well plates. **b** A representative microscopic view of von Kossa method staining at a magnification of ×400 in 24-well plates. **c** Calcium content was reduced by apelin (*P < 0.05 vs. PCM group)

After stimulating the cells for 14 and 21 days, ALP expression and cell mineralization were strongest (Figs. 2, 3, 4). Figures 2 and 3 show cells treated with normal medium, PCM, and PCM with apelin at 10 pM, 100 pM, 1 nM and 10 nM every other day. In comparison with the blank control and the PCM groups, western blotting showed that apelin decreased the ALP activity and expression dramatically in a dose-dependent manner (Figs. 2a, b, 3) (*P < 0.05 vs. PCM), especially at the concentration of 100 pM. However, this effect was suppressed by PD98059 and LY29402 (^#^*P* < 0.05 vs. PCM + 100 pM apelin). Figure 4 shows the result for cells treated with normal medium, PCM and PCM with 100 pM apelin for 21 days every other day. The cell calcification was most obvious in the PCM group, and it was significantly attenuated by apelin in the form of calcium content (*P < 0.05 vs. PCM).

**Fig. 5:**
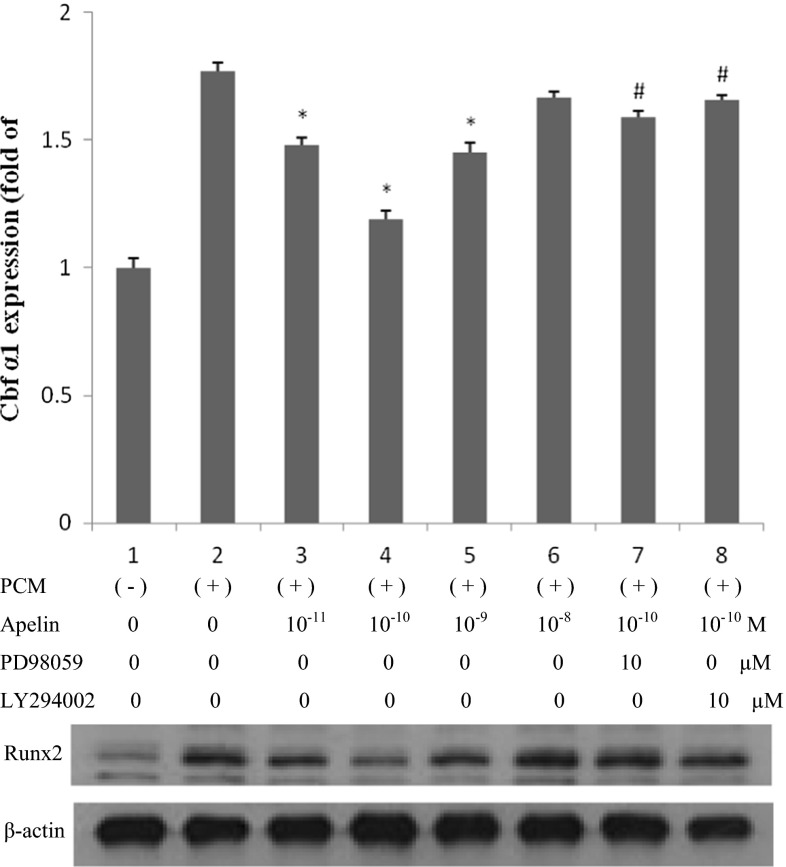
The effect of apelin on Runx2 expression. Human AVICs were induced by PCM with apelin of different concentrations (0, 10 pM, 100 pM, 1 nM and 10 nM) as well as PD98059 (ERK inhibitor) and LY29402 (Akt inhibitor) for 24 h

Similarly, we stimulated cells with the above supplement for 24 h, and Runx2 expression was examined. In Fig. 5, the changes in Runx2 expression were consistent with ALP and mineralization, and the apparent decrease occurred at 100 pM apelin. Western blotting showed that apelin dramatically decreased Runx2 expression in a dose-dependent manner (*P < 0.05 vs. PCM), especially at 100 pM (*P < 0.05 vs. PCM + other concentration groups of apelin). This effect was suppressed by PD98059 and LY29402 (^#^*P* < 0.05 vs. PCM + 100 pM apelin).

### Apelin activates the ERK and PI3-K/Akt pathways in human AVICs

**Fig. 6:**
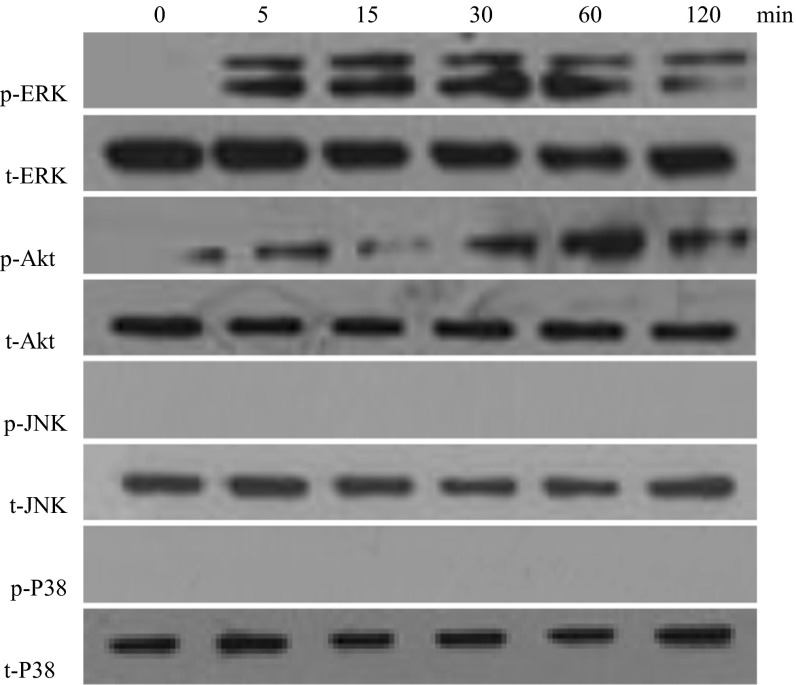
The effect of apelin on MAKP and PI3-K/Akt activation in the osteoblastic differentiation of human AVICs. AVICs were incubated with 100 pM apelin in PCM for 0, 5, 15, 30, 60 or 120 min after incubation in serum-free medium for 5 h. Cell lysates were subjected to western blotting and incubated with antibodies against p-ERK, t-ERK, p-Akt, t-Akt, p-JNK, t-JNK, p-P38, and t-P38. The results show that exposure of the cells to 100 pM apelin for 5–120 min activated the ERK and Akt signaling pathways

In our previous work, mitogen-activated protein kinase (MAPK) and PI3-K/Akt were found to play essential roles in regulating cell differentiation and it was determined that apelin regulates osteoblastic differentiation through the ERK and PI3-K/Akt pathways (Shan et al. 2011). Hence, we examined the level of MAPK and PI3-K/Akt signaling, induced by apelin (100 pM) with PCM in human AVICs, at 0, 5, 15, 30, 60 and 120 min (Fig. 6). Phosphorylated c-Jun N-terminal kinases (p-JNK) and phosphorylated p38 MAP kinases were not detected at any time; however, phosphorylated ERK (p-ERK) and phosphorylated Akt (p-Akt) increased dramatically after 5 min of incubation. This result indicates that ERK and PI3-K/Akt can be activated by apelin. This was further confirmed by treating the cells with PD98059 (ERK inhibitor) and LY294002 (PI3-K/Akt inhibitor). As shown in Figs. 3 and 5, ALP and Runx2 expression did not decrease.

## Discussion

Ectopic calcification of the cardiovascular system predominantly affects the aorta, coronary arteries and peripheral arteries. Traditionally, cardiovascular calcification has been considered to be passive phenomenon associated with aging. However, it is currently viewed as an actively regulated disease process, and increasing evidence suggests that the underlying mechanisms of cardiovascular calcification are similar to embryonic bone formation (Demer and Tintut 2008; Otto 2008; Towler and Demer 2011). AVC is considered as similar to osteogenesis. AVICs may undergo a phenotype transition to become osteoblast-like cells and elaborate bone matrix (Leopold 2012; Rajamannan et al. 2003). AVICs are the predominant cells in the aortic valve, and aortic valve disease is usually related to AVICs lesions. Therefore, the transition process of AVICs to osteogenic phenotype may play a central role in AVC. The process is similar to that in VSMCs during arterial calcification (Feng et al. 2012; Liao et al. 2008).

The transition of AVICs to an osteogenic phenotype usually occurs in a specific pro-calcific matrix (PCM). β-GP is the major pro-calcific component, and DXM and ascorbic acid should also be supportive. We have successfully established a model of AVICs calcification in vitro (Feng et al. 2012). The present study further supports the cell culture calcification model. Of the two chosen markers, ALP is an early marker of osteoblastic differentiation and Runx2 is the nuclear transcription factor of osteoblastic differentiation which binds to the osteoblast-specific cis-acting element OSE2 (Liao et al. 2013; Liu et al. 2014).

Apelin is an adipokine that distributes primarily in the endothelium and activates APJ via both pericellular and endocrine signaling pathways. In the cardiovascular system, APJ is expressed widely by myocardial cells, endothelium and VSMCs. Increasing evidences have demonstrated that the apelin/APJ system plays a critical role in regulating cardiovascular function and in mediating adaptation to physiological stress and diseases. This system is involved in lowering arterial blood pressure, endothelium-intact mammary artery vasodilatation, facilitating neovascularization in the peri-infarct myocardium, decreasing systemic venous tone, reducing left ventricular preload and afterload without cardiac hypertrophy, promoting dieresis and contractility in cardiomyocytes (Farkasfalvi et al. 2007). Moreover, apelin/APJ has been found to correlate with many cardiovascular diseases, such as atherosclerosis, coronary heart disease, heart failure, hypertension, pulmonary artery hypertension, myocardial ischemia–reperfusion injury and atrial fibrillation (Yu et al. 2014). Recently, the apelin/APJ signaling pathway was found to be significantly up-regulated in calcific aortic valves, but the exact ectopic calcific mechanism was not elucidated (Peltonen et al. 2009). However, some studies have shown that apelin/APJ can suppress osteoblastic differentiation of VSMCs via the ERK and PI3-K signaling pathways in vitro (Shan et al. 2011).

The present research demonstrates that apelin/APJ inhibits the osteoblastic differentiation of AVICs in a dose-dependent manner in cell culture. Interestingly, the optimal protective effect occurs at the concentration of 100 pM apelin other than 1 or 10 nM. This phenomenon is consistent with the result of apelin inhibiting osteoblastic differentiation of vascular smooth muscle cells (Shan et al. 2011), which showed that effect of apelin occurred dramatically at 1 nM other than 10 nM. Given these findings, we conclude that apelin plays a widely part in variety of tissues calcification, and the effect of apelin would take the concentration into account. The mechanism involves activation of the APJ/ERK and APJ/PI3-K/Akt signaling pathways. Apelin appears to have a protective role in AVC and it is possible that treatment with apelin could be helpful in prevention of AVC.

## Abbreviations

ALP: Alkaline phosphatase
AVC: Aortic valve calcification
AVICs: Aortic valve interstitial cells
AS: Aortic valve stenosis
BCIP/NBT: 4-Chloro-3-indolyl phosphate/nitroblue tetrazolium chloride
CAVD: Calcific aortic valve diseases
CAVICs: Calcific human AVICs
Runx2: Runt-related transcription factor 2
DXM: Dexamethasone
ERK: Extracellular signal-regulated kinase
β-GP: Beta-glycerophosphate disodium
MAPK: Mitogen-activated protein kinase
PBS: Phosphate buffer saline
PCM: Pro-calcific medium
PI3-K: Phosphatidylinositol 3-kinase
VHD: Valvular heart diseases
Vit C: Ascorbic acid
VSMCs: Vascular smooth muscle cells
